# MicroRNA-450a-3p Represses Cell Proliferation and Regulates Embryo Development by Regulating Bub1 Expression in Mouse

**DOI:** 10.1371/journal.pone.0047914

**Published:** 2012-10-22

**Authors:** Min Luo, Yaguang Weng, Jian Tang, Min Hu, Qiang Liu, Fengbing Jiang, Dandan Yang, Chen Liu, Xiaoqin Zhan, Peipei Song, Huili Bai, Baolin Li, Qiong Shi

**Affiliations:** 1 Key Laboratory for Clinical Laboratory Diagnostics of Education Ministry of China and School of Clinical Diagnostic and Laboratory Medicine, Chongqing Medical University, Chongqing, People's Republic of China; 2 The Affiliated Chengdu Women and Children's Central Hospital of Chongqing Medical University, Chengdu, People's Republic of China; University of Hawaii Cancer Center, United States of America

## Abstract

Bub1 is a critical component of the spindle assembly checkpoint (SAC) and closely linked to cell proliferation and differentiation. We previously found that spontaneous abortion embryos contained a low level of Bub1 protein but normal mRNA level, while the knockdown of Bub1 leads to abnormal numerical chromosomes in embryonic cells. Here, we investigated the mechanism through which governs the post-transcriptional regulation of Bub1 protein expression level. We first conducted bioinformatics analysis and identified eight putative miRNAs that may target Bub1. Luciferase reporter assay confirmed that miR-450a-3p can directly regulate Bub1 by binding to the 3′-untranslated region of Bub1 mRNA. We found that the overexpression of miR-450a-3p in mouse embryonic fibroblast (MEF) cells down-regulated Bub1 protein level, repressed cell proliferation, increased apoptosis and restricted most cells in G1 phase of the cell cycle. Furthermore, when the fertilized eggs were microinjected with miR-450a-3p mimics, the cleavage of zygotes was effectively suppressed. Our results strongly suggest that an abnormally decreased Bub1 level regulated by miRNAs may be implicated in the pathogenesis of spontaneous miscarriage. Therefore, the blockade of miR-450a-3p may be explored as a novel therapeutic strategy for preventing spontaneous miscarriages.

## Introduction

Accurate segregation of chromosomes during mitosis is essential to maintain genomic integrity[1]. To ensure genome stability, eukaryotic cells have developed an inhibitory signaling network commonly referred to as the spindle assembly checkpoint (SAC), which can delay anaphase onset until all the sister kinetochores of duplicated chromosomes are properly aligned and stably attached to microtubules emanating from opposite spindle poles[2], [3]. Abnormal chromosome segregations may lead to preternatural numbers of chromosomes, and even provoke cell cycle arrest [1], [4], [5]. Bub1 is a critical component of the SAC. As the “sensor” protein of SAC surveillance mechanism, Bub1 is known to regulate cell proliferation and differentiation [6], [7], [8]. Homozygous Bub1-null mice died shortly after E3.5 [8], [9]. Bub1 is essential for the spindle checkpoint response, and also for the correct alignment of chromosomes on the metaphase spindles [10].

In adult males tamoxifen-induced inactivation of Bub1 impairs normal chromosome segregation and inhibits spermatogenesis, which may lead to infertility. Bub1 is also critical for the post-implantation development [1]. Bub1 is associated with self-renewal and pluripotent differentiation in embryonic stem cells [11]. Wells et al reported that the Bub1 expression is low in 2-cell embryos, but is significantly up-regulated in hatched blastocysts, indicating that the low level of Bub1 may be important for maintaining the stem cell properties prior to embryo implantation [12]. We previously found that the knockdown of Bub1 led to abnormal chromosomes in embryonic cells, and that the expression of Bub1 was significantly reduced and the numbers of spontaneous abortion embryo samples with aberrant numerical chromosome were increased [13]. However, it is unclear how Bub1 expression is regulated in this process.

In our previous study, spontaneous abortion embryos contained low level of Bub1 protein but normal mRNA expression, indicating that the Bub1 expression may be regulated at post-transcriptional level. A vast post-transcriptional regulatory network is mediated by miRNAs which regulate gene expression through at least two distinct mechanisms: mRNA degradation and mRNA translational repression [14], [15], [16]. They interact with mRNA through imperfect or perfect base pairing in the 3′-untranslated region, resulting in translational repression or m RNA destabilization and degradation [15], [17]. It has been shown that microRNAs function as important regulators of embryonic stem cell differentiation, limb development, adipogenesis, myogenesis, angiogenesis and hematopoiesis, neurogenesis, and epithelial morphogenesis[18]. It is estimated that miRNA targets more than 5300 human genes [19]. Knockout of Dicer results in embryonic death before E7.5, indicating that miRNAs are crucial for mouse development [20]. Given the fact that homozygous Bub1-null mice died shortly after E3.5 [8], [9], these findings suggest that miRNAs may target Bub1 during embryonic development and may cause abnormal low level of Bub1, leading to pathological conditions, such as spontaneous miscarriages.

In this study, we first conduct bioinformatics analysis and identify eight potential miRNAs that may target Bub1. Among them, miR-450a-3p is confirmed to directly target Bub1. We further reveal that miR-450a-3p suppresses cell proliferation and impairs cell cycle progression. When the fertilized eggs are microinjected with miR-450a-3p mimics, the cleavage of zygotes is effectively suppressed. Our findings strongly suggest that miRNA-mediated targeting of Bub1 expression by miRNA may be implicated in the pathogenesis of spontaneous miscarriage.

## Results

### The 3′-UTR of Bub1 is targeted by miR-450a-3p

**Figure 1:**
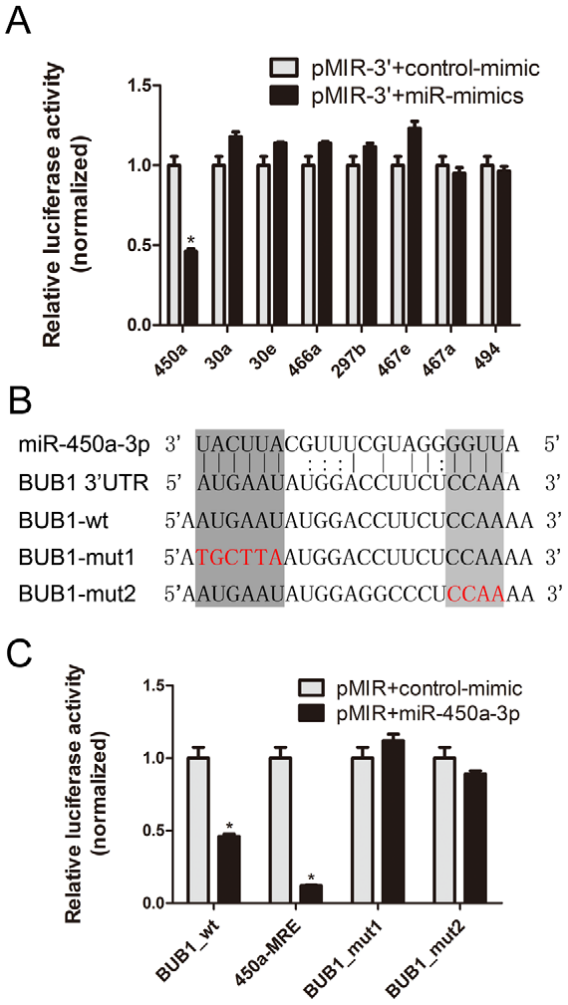
miR-450a-3p binds to the 3′ UTR of mouse Bub1 mRNAs. (**A**) pMIR-3′ vector contains Bub1 mRNA 3′UTR and miRNA-mimics or control mimic were co-transfected into HEK 293 cells, Cells lysates were prepared after 48 h for measuring luciferase activity, which was normalized to β-galactosidase expressions. The suppression of luciferase activity was induced only by Ad-miR-450a-3p. Data are shown as the mean±SEM of three replicates and are representative of three independent experiments (*p<0.001). (**B**) Sequence of the miR-450a-3p binding sites within the mouse Bub1 mRNA. Schematic of the pMIR-reporter constructs showed the wild type 3e prepared after 48 h for measurement and the mutated 3′UTR nucleotides (red) of the miR-450a-3p binding site (Bub1mut1 and Bub1 mut2). (**C**) Luciferase activity of the Bub1_wt (pMIR-3′) and the 450a_MRE (reporter containing a perfect complementary of miR-450a-3p) in the presence of miR-450a-3p -mimic showed the marked inhibitory activity of these two reporters and the Bub1 mut1 and mut2 reporters, showed that miR-450a-3p-mimic cannot inhibit the luciferase activity of these constructs compared with the wild-type construct. Error bars (SEM) are derived from three experiments in triplicate (means±SEM, n = 3; *p<0.001).

Two different algorithms (TargetScan and miRanda) were used to identify putative miRNAs that could bind to the 3′-UTR of Bub1. Eight potential microRNAs were identified, including miRNA-30a,30e,494,467a,467e,450a-3p,466a-3p and 297b. In order to determine whether the 3′-UTR region of Bub1 mRNA is a direct functional target of predicted microRNAs, we cloned a 191-bp fragment of the mouse Bub1 3′-UTR harboring the potential binding site into downstream of the pMIR-REPORT vector to generate the pMIR-3′ vector. A β-gal vector was used to normalize the transfection efficiency. These vectors and miRNA-mimics were co-transfected into HEK 293 cells. At 48 h the luciferase activity was significantly suppressed by 54% (p<0.001) in HEK 293 cells treated with pMIR-3′ vector and miR-450a-3p-mimic, compared with control (**Fig. 1A**). No repression of luciferase activity was observed in other groups. Computational analysis predicted that the 3′-UTR of Bub1 transcript contains a 14-mer miR-450a-3p nucleotides complementation after the stop codon (**Fig. 1B**). This suggests that miR-450a-3p is likely to be a regulator of Bub1 through targeting the Bub1 3′-UTR.

In an effort to confirm that the Bub1 3′-UTR is a target for miR-450a-3p, we transfected into HEK 293 cells a luciferase reporter containing a perfect complementary MRE (miRNA response element, MRE) of miR-450a-3p to provide a site with high binding affinity for the miRNA. Co-transfection of the cells with 450a-MRE suppressed the luciferase activity by ∼90%, while no suppression was observed with the control miR-mimic (**Fig. 1C**). To validate this result, we mutated the miR-450a-3p binding site on Bub1 3′-UTR (**Fig. 1B** **, Bub1 mut1 and mut2**). The mutations prevented miR-450a-3p from interfering with luciferase activity. These results indicate that the 3′-UTR of Bub1 is likely a target of miR-450a-3p (**Fig. 1C**).

### The miR-450a-3p down-regulates Bub1 expression at the transcriptional level

**Figure 2:**
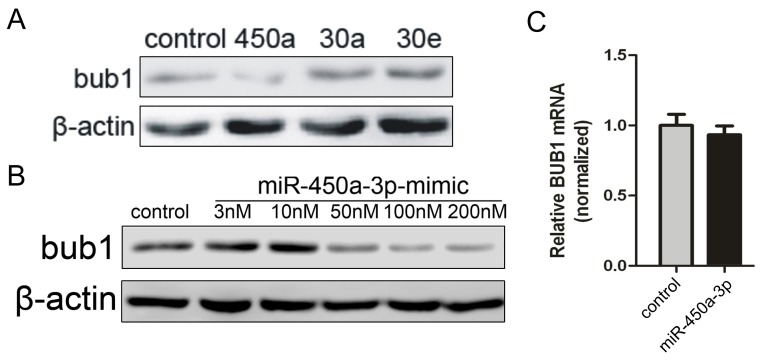
miR-450a-3p leads to down regulation of Bub1 protein. (**A**) iMEFs were treated with 50 nM miRNA-mimic or 50 nM control mimic for 48 h. Cells were harvested and subjected to western blot analysis using anti-mouse Bub1 antibody and anti-β-actin antibody was used as a loading control. The results are representative of three independent experiments. (**B**) iMEFs were transfected with 200 nM control mimic or increasing amounts (3 nM, 10 nM, 50 nM, 100 nM, 200 nM) of miR-450a-3p-mimic. Forty-eight hours after mimic transfection, cells were harvested and subjected to western blot analysis. The experiment was repeated twice, with similar results. (**C**) iMEFs were transfected with 50 nM control mimic or 50 nM miR-450a-3p mimic. Twenty-four hours after mimic transfection, cells were harvested and subjected to qRT–PCR analysis. Relative Bub1 mRNA level normalized to GAPDH is plotted as means±SEM, n = 3; *p* = 0.7.

We next tested if ectopic expression of miR-450a-3p would reduce Bub1 protein level in iMEF cells. We found that treatment of iMEF cells with miR-450a-3p mimic (50 nM) positively caused a marked reduction of Bub1 expression, whereas treatment with miR-30a or 30e mimic or an negative control (non-targeting) mimic did not cause any reduction of Bub1 protein (**Fig. 2A**). It was reported that miRNA expression levels and target mRNA suppression may depend on a threshold of miRNA concentration [21] and increasing amounts of miRNA mimic induce different extent reducing of target mRNA expression [22]. We tested five doses of chemically synthetic miR-450a-3p-mimic in iMEFs, followed by Western blot analysis of Bub1 protein. Various concentrations of miR-450a-3p mimic (3 nM, 10 nM, 50 nM, 100 nM, 200 nM) were transfected into iMEFs (**Fig. 2B**). We found that the level of Bub1 protein level did not change when the mimic concentration was 3 nM or 10 nM. However, higher concentrations (e.g., 50 nM) of miR-450a-3p-mimic significantly reduced Bub1 protein to a lower level than that in iMEFs with the negative control (**Fig. 2B**). Interestingly, the levels of Bub1 protein did not significantly decrease with the higher concentration of miR-450-3p mimic (**Fig. 2B**). Nonetheless, these findings indicate that Bub1 can be negatively regulated by miR-450a-3p in iMEF cells.

To examine whether miR-450a-3p down-regulates the mRNA expression of Bub1 in iMEFs, miR-450a-3p-mimic was transfected into iMEFs, followed by qRT–PCR analysis of Bub1 mRNA. Under these conditions, the expression of Bub1 mRNA did not change (**Fig. 2C**), suggesting that miR-450a may target Bub1 mRNA through its suppression rather than its degradation.

### miR-450a-3p suppresses iMEF cell proliferation

**Figure 3:**
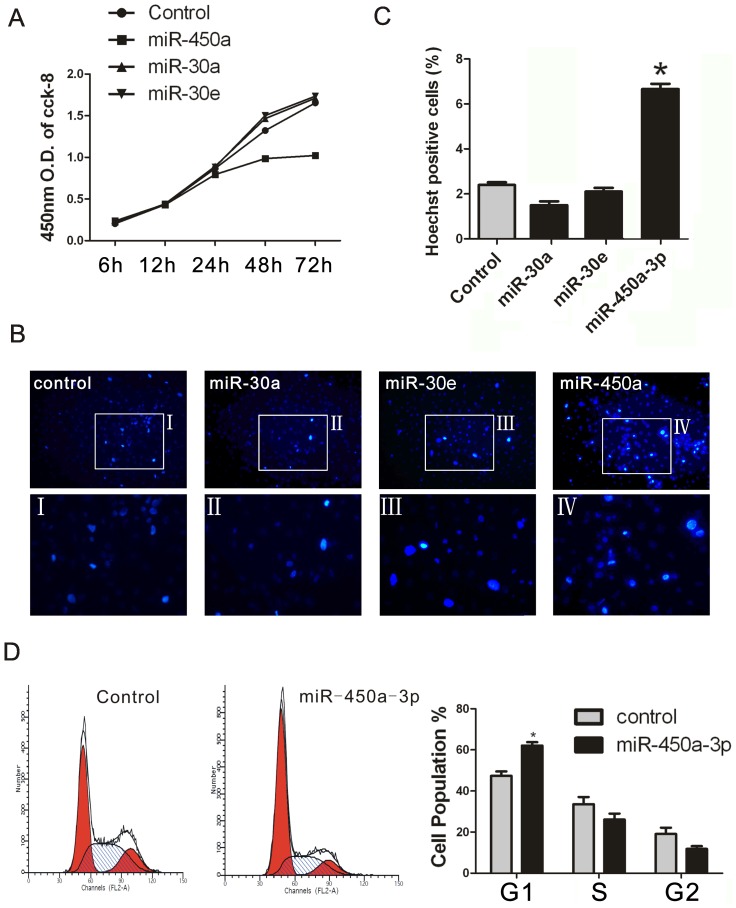
Effect of miR-450a-3p overexpression on proliferation and apoptosis in iMEFs cells. (**A**) iMEF cells were transfected with miRNA-mimic or negative control. Cells were harvested at various time points (6 h, 12 h, 24 h, 48 h, 72 h), proliferation was measured using the Cell Counting Kit-8. This experiment was performed four times, with similar results each time. (**B**) Transfected cells were measured using the Hoechst 33258 after 48 h. Graphs show representative results from one of three independent experiments. (**C**) In each repetition, 300 cells were counted to calculate the Hoechst positive cell percentage. The data are presented as the mean±SEM. (n = 3; * *p*<0.05). (**D**) Transfected cells were harvested after 48 h, fixed with 70% ethanol and treated with RNase. After PI staining, cells were analyzed by flow cytometry. Representative results from one of three independent experiments are presented. Data are the mean of three independent experiments, presented as mean±SEM. (n = 3; *p<0.05 was calculated using G1 population by independent samples t test).

Bub1 is a protein kinase that involves in spindle checkpoint response [23] and controls the checkpoint [24]. To investigate the function of miR-450a-3p, iMEF cells are transfected with miRNA mimic or negative control. Cell proliferation was measured by the Cell Counting Kit-8. We found that over-expression of miR-450a-3p repressed cell proliferation and there was a nearly 40% reduction of proliferation after transfection at 72 h (**Fig. 3A**).

To determine whether the reduced cell proliferation was apoptosis-independent, we performed Hoechst staining to detect apoptosis. iMEFs were transfected with miRNA mimic or negative control. After 48 h, the numbers of Hoechst-positive cells treated with miR-450a-3p were more than that treated with control mimic (**Fig. 3B**). The percent of Hoechst positive cells treated with miR-450a-3p is 6.8%, while the percent of Hoechst positive cells treated with miR-30a, miR-30e and the control is 2.4%, 1.5%, and 2.1% respectively (**Fig. 3C**). However, the percentage of the Hoechst positive cells with miR-450a-3p was low, which may at least partially explain the marked inhibition of proliferation caused by miR-450a-3p expression may be not entirely caused by apoptosis. Flow cytometry analysis was performed to examine whether miR-450a-3p alters the cell cycle progression. After transfected with miR-450a-3p mimic or control mimic for 48 hours, iMEF cells were harvested and stained with Propidium Iodide (PI). The results of flow cytometry analysis confirmed that cells treated with miR-450a-3p were mostly arrested at the G1 phase. Overexpression of miR-450a-3p increased the fraction of cells in the G1 phase from 43.34% to 62.09% while decreasing the fraction of cells in the G2/M phase from 19.08% to 11.82% (**Fig. 3D**). These results suggest that miR-450a-3p may repress cell proliferation by arresting cells in the G1 phase. Thus, down-regulation of Bub1 in cells would lead to dysfunction of spindle assembly checkpoint and more cells arrested in the G1 phase.

### Overexpression of Bub1 reduces the proliferation inhibition caused by miR-450a-3p

**Figure 4:**
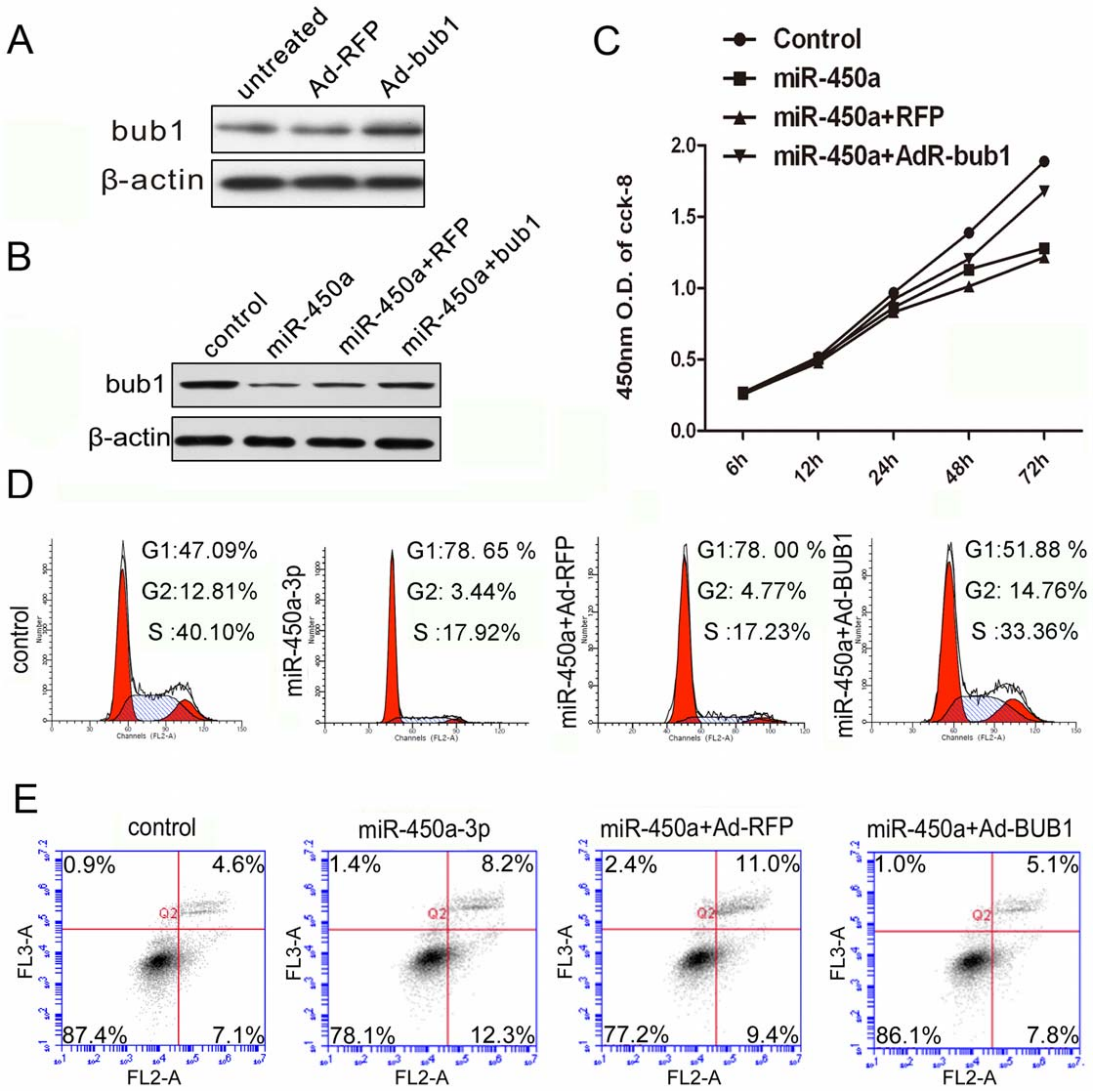
Bub1 is required for miR-450a-directed iMEFs proliferation inhibition. (**A**) iMEF cells were infected with Ad-Bub1 or RFP for 48 h. Cells were harvested and subjected to western blot analysis. (**B**) Immunoblotting of Bub1 in extracts from iMEF cells that were infected with Ad-Bub1 and transfected with miRNA-450a-3p mimic both or alone for four different treatments: (i) control-mimic; (ii) miRNA-450a-3p-mimic; (iii) miRNA-450a-3p and Ad-RFP; (iv) miRNA-450a-3p-mimic and Ad-Bub1. (**C**) Cell proliferation analysis of iMEFs that were treated with four different methods, the same as those used in Fig.5B. (**D**) Cell cycle analysis of iMEFs that were treated as described in B. (**E**) Cell apoptosis analysis of iMEFs that were treated as described in B. All the results are representative of three independent experiments.

We next examined the functional relevance of the miR-450a-3p/Bub1 interaction in mouse embryo fibroblasts. The iMEF cells were infected with Bub1 adenoviral vector (Ad-Bub1) or a control vector (Ad-RFP) , and then transfected with or without miRNA-450a-3p mimic. We confirmed that Ad-Bub1 effectively expressed Bub1 at protein level (**Fig. 4A**). Bub1 protein level was higher in cells transduced with miRNA-450a-3p-mimic and Ad-Bub1 than that in the cells transduced with miRNA-450a-3p-mimic and Ad-RFP (**Fig. 4B**). Thus, down-regulation of Bub1 by the introduction of miR-450a-3p-mimic was successfully rescued by Ad-Bub1 infection. The proliferation rate was higher in cells co-transduced with miRNA-450a-3p and Ad-Bub1 (**Fig. 4C**). Bub1 rescue in miRNA-450a-3p treated cells led to an increase in cell numbers in S phase and a decrease of cell apoptosis (**Fig. 4D and 4E**). These findings suggest that the repression of cell proliferation by miR-450a may be caused by the down-regulation of Bub1.

### Microinjection of miR-450a-3p suppresses embryo development

**Figure 5:**
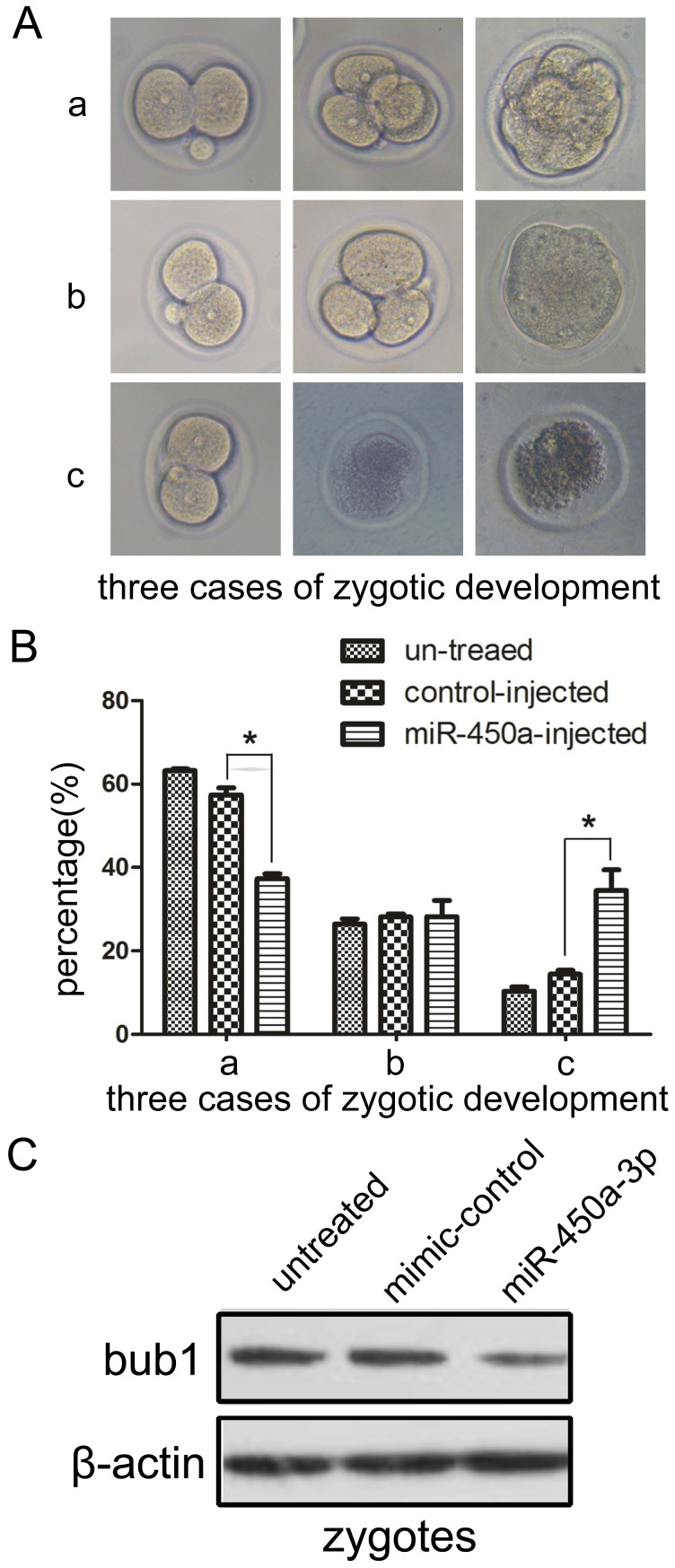
Effects of miR-450a-3p on zygote cleavage. (**A**) There are three cases of zygotic development when 2-cell embryos received microinjections of miR-450a-3p or control mimic: ‘a’ denotes zygotes that developed to the 8-cell embryo; ‘b’ denotes zygotes that developed to the 4-cell embryo but failed to reach the 8-cell stage; ‘c’ denotes zygotes that failed to reach the 4-cell embryo stage. (**B**) Microinjection of miR-450a-3p suppressed the development of 2-cell embryo. Three cases of zygotic development percentage were based on the number of 2-cell embryos used. Statistically significant differences (* *p*<0.05) were found between the miR-450a-3p-injected embryos and the control-injected embryos. There was no significant difference between untreated embryos and control-injected embryos. The experiment was repeated 3 times, and at least 100 embryos were involved in each treatment. (**C**) Western blot analysis of Bub1 protein from embryos. There was a significant decrease in Bub1 protein expression in embryos microinjected with miR-450a-3p relative to the mimic control or the untreated control. Each lane contained extract from 40 embryos.

We further investigated the effect of miR-450a-3p on embryo development. Microinjection of miR-450a-3p-mimic into the two-cell embryos was carried out. Over-expression of miR-450a-3p significantly reduced cell divisions of zygotes when compared to those injected with scramble control. About 28.26% of the miR-450a-3p-injected zygotes failed to develop into the 8-cell stage and 34.47% failed to develop into the 4-cell, while 28.15% of the scramble control-injected zygotes failed to develop into the 8-cell stage and 14.42% failed to develop into the 4-cell stage (**Fig. 5A and 5B**). There was no significant difference in the embryo development of the untreated (without injection) and those injected with control mimic. The expression of Bub1 in miR-450a-3p treated zygotes was also confirmed (**Fig. 5C**). Taken together, these results strongly suggest that miR-450a-3p may play an important role in regulating Bub1 level and a abnormally low level of Bub1 may lead to spontaneous miscarriage.

## Discussion

The discovery and functional studies of miRNAs was one of the significant progresses in life sciences. In this study, we focused on a well-known spindle assembly checkpoint protein Bub1 and validated it as a direct target of miR-450a-3p. The mechanism by which miR-450a-3p negatively regulates Bub1 is translational repression through imperfect base pairing with the 3′-UTR. A common mechanism in regulating gene expression in eukaryotes relies on 3′-UTR-mediated post-transcriptional control of maternal genes [25]. It has been shown that miR-10a binds the 5′-UTR of ribosomal protein mRNAs and enhances their translation rather than negative regulation [26]. It was also shown that miR-184 can interfere with the ability of miR-205 to suppress SHIP2 level, indicating that one miRNA could abrogate the inhibitory function of another [27]. All the findings indicate that regulatory network of miRNA is complex and may cross-talk with other genes.

Our results have demonstrated that overexpression of miR-450a-3p represses iMEF cell proliferation and down-regulates Bub1 protein level. Consistent with the findings reported by Draviam et al, MEFs can undergo marked reduction in cell numbers and highly aberrant mitosis in the absence of Bub1[1]. Anti-proliferative effect observed in iMEFs treated with miR-450a-3p is less obvious than which in Bub1-null (repressing Bub1 by ∼98%) primary embryonic fibroblasts. However, the extent of inhibition of Bub1 by miR-450a-3p is much less than that through gene knockout technology. Partial inhibition of Bub1 may account for this difference. We also find that overexpression of miR-450a-3p can increase apoptosis in iMEF cells, which is supported by other reports. For example, depletion of hBub1 enhances p53-dependent early apoptosis in HCT116 cells [28]. Homozygous Bub1 mutant alleles in mouse embryo fibroblasts cause premature senescence in the presence of p53 [29]. Bub1 insufficiency mediates cell death in response to chromosome missegregation [30]. Therefore, it is not surprising that microinjection of miR-450a-3p can suppress zygote cleavage.

In summary, we find that overexpression of miR-450a-3p can induce Bub1 down-regulation, leading to the inhibition of cell proliferation, an increase in apoptosis and restriction of most cells in the G1 phase. We also demonstrate that miR-450a-3p is involved in embryo development. The action of miR-450a-3p is at least in part through down-regulation of Bub1. We previously found that the decreased expression of Bub1 proteins is associated with spontaneous miscarriages [13]. It is known that the impairment of the mitotic spindle checkpoint may play a major role in the cancer formation [31], [32]. Thus, as Bub1 is an important component of the spindle assembly checkpoint, it is conceivable that its abnormal expression may lead to developmental abnormalities and possible spontaneous miscarriage. Our result shows that miR-450a-3p targets mice Bub1 mRNA 3′-UTR and down-regulate Bub1 protein. Thus, the blockade of miR-450a-3p may constitute a novel therapeutic strategy for preventing spontaneous miscarriages.

## Materials and Methods

### Cell Culture

HEK-293 cells and iMEF cells were cultured in Dulbecco's Modified Eagle's Medium (DMEM high glucose) containing 10% fetal bovine serum (Hyclone) and 100 units/ml penicillin (Invitrogen), and 100 g/ml streptomycin (Invitrogen). All cells were incubated in a humidified atmosphere of 5% CO_2_ at 37°C.

### MicroRNA and Bub1 mRNA qRT–PCR

iMEFs cells were transfected with miRNA-mimics and harvested after 48 h. All samples were transported to RIBOBIO Company at low temperature for qRT–PCR (SYBR Green) analysis of mature miRNA. The relative expression was calculated using the ΔΔCT method and normalized to the expression of U6. The levels of Bub1 mRNA in iMEFs were examined by SYBR green real-time quantitative reverse transcription-PCR (qRT-PCR) (Applied Biosystems, USA) and normalized to GAPDH mRNA. The primers for Bub1 were: forward primer 5′-TGGTTGAACAAGTCCACAGC-3′,reverse primer 5′- CTGACCCAGGTCAATCAATG-3′. The annealing temperature was 59°C. All qRT-PCRs were performed in duplicate.

### Vector Construction and Reporter Assays

The mouse Bub1 mRNA 3′UTR was cloned between the SpeI and HindIII sites in the pMIR-Report. Bub1mutant sequences were cloned from the Bub13′UTR pMIR-Report using the mutant primer, as follows:

F1:5′-GAAAGCTGCGCACTAGTATGCTTAATGGACCTTCTCCAAAAC-3′;R1:5′-GTTTTGGAGAAGGTCCATTAAGCATACTAGTGCGCAGCTTTC-3′;F2:5′-CGCACTAGTAATGAATATGGAGGCCCTCCAAAACACACTTAG-3′;R2:5′-CTAAGTGTGTTTTGGAGGGCCTCCATATTCATTACTAGTGCG3′. The miR-450a-3p complementary luciferase vector was constructed by inserting an oligo containing two consecutive perfect matches to miR-450a-3p to the luciferase gene in the pMIR-Report. The primer sequences were as follows: F: 5′-CTAGTTAACCCCTACGAAACGTAAGTAA-3; R: 5′AGCTTTACTTACGTTTCGTAGGGGTTAA-3′. All the primers were purchased from TaKaRa. iMEFs cells and HEK293 cells were seeded onto 24-well plates (1×105 cells per well) the day before transfections were performed. Cells (≈70% confluent) were transfected with 50 ng pMIR-Report (Ambion) constructs per well, 50 nM miR-mimics (RIBOBIO), and 100 ng β-gal vector using Lipofectamine 2000 (Invitrogen). Cell lysates were prepared with Passive Lysis Buffer (Promega) 48 h after transfection, and luciferase activities were measured by using the Dual Luciferase Reporter Assay (Promega). β-galactosidase expression was measured by β-galactosidase Assay Kit (Beyotime). β-gal provides an internal control that serves as the baseline response.

### Western blot analysis

Cell lysates were prepared in lysis buffer following 48 h transfection with either different concentration miR-450a-3p-mimic or control mimic. 50 µg of total cell lysates per lane was separated by 8% SDS-PAGE. For examining the expression of Bub1 in embryos, zygotes microinjected with miR-450a-3p or control mimic were collected, rinsed twice in PBS, resuspended in 10 ml of Laemmli buffer and boiled for 10 minutes. Each lane represented a pool of 40 embryos. Immunoblotting was performed with mouse anti-Bub1 (1∶200; Abcam), and mouse anti-β-actin (1∶1000; Santa) primary antibodies. Membranes were subsequently probed with a secondary antibody (1∶5000; Zhongshan). Antigen-antibody complexes were visualized using enhanced chemiluminescence.

### Proliferation, Apoptosis, and Cell Cycle Assays

Cells were seeded in 24-well plates at ∼0.5×105 cells per well the day before transfection. iMEFs cells were transfected with 50 nM miRNA-mimic or negative control. Proliferation was measured using the Cell Counting Kit-8 (DojinDo, Japan) following the manufacturer's instructions. Briefly, cells were harvested at various time points. The cell suspension (500 µl/well) was inoculated into a 24-well plate. CCK-8 solution (50 µl) was add to each well of the plate. The plates were incubated for 2 hours. The absorbance was measured at 450 nm using an ELIASA reader.

To measure cell apoptosis, the proportion of iMEFs cells undergoing apoptosis were measured using Hoechst 33258 (Beyotime). Briefly, cells were washed twice with cold PBS, and the Hoechst 33258 solution (10 µg/ml) was added. Cells were incubated for 20 minutes at 37°C in the dark. Cells positive for the dye were observed using ultraviolet light. A second cell apoptosis assay, PE Annexin V Apoptosis Detection Kit I (BD Biosciences), was used. After staining with PE Annexin V and 7-AAD, the samples were analyzed within 1 hour using flow cytometry.

For cell cycle analysis, samples were harvested 48 hours post transfection, washed twice with cold phosphate-buffered saline and fixed with 70%, ethanol at 4°for more than 12 h and then added RNase A 200 µl/ml 37°for 1 h. Cells were analyzed using flow cytometry. Results are presented as the percent of the cell population in each cell cycle phase. Data are the mean of three independent experiments±SEM. The *p* value was calculated using G1 population by an independent sample t test.

### Mouse embryo collection, culture in vitro and microinjection

The protocol of this study was approved by the Committee on Use of Live Animals in Chongqing Medical University. ICR female mice aged 6–8 weeks were superovulated by consecutive injections of 5 IU of pregnant mare serum gonadotropin (Sigma) and 5 IU of human chorionic gonadotropin (hCG, Sigma) 47 to 48 hours apart. Fertilized zygotes at the 2-cell stage were flushed out from the oviducts at 40 to 42 hours after the hCG injection. About 10 pL of 25 mM miR-450a-3p-mimic was microinjected into the zygotes' cytoplasm. Control-mimic injected embryos were used as control for assessing injection damage. After microinjection, groups of approximately 20 embryos were cultured in 50 ml of M16 MODIF (Millipore) medium supplemented with 10% fetal bovine serum (Hyclone) and overlaid with mineral oil at a humidified atmosphere of 5% CO_2_ at 37°C. Embryo development was observed under an inverted microscope.

### Statistical analysis

Statistical analysis was performed using the SPSS13.0 software. Values are expressed as the mean±SEM. Differences between groups were calculated with Student's test. A *p*<0.05 was defined as statistically significant.
